# Hospital-based prostate cancer screening in vietnamese men with lower urinary tract symptoms: a classification and regression tree model

**DOI:** 10.1186/s12894-022-01116-2

**Published:** 2022-10-29

**Authors:** Nguyen Chi Cuong, Nguyen Truong Vien, Nguyen Minh Thien, Phan Thanh Hai, Tran Ngoc Dang

**Affiliations:** 1 Ho Chi Minh City MEDIC Medical Center, Ho Chi Minh City, Vietnam; 2 Faculty of Public Health, University of Medicine and Pharmacy at Ho Chi Minh City, Ho Chi Minh City, Vietnam; 3 Pham Ngoc Thach University of Medicine, Ho Chi Minh City, Vietnam

**Keywords:** Prostate cancer, PSA, I-PSS, Vietnamese patients, Classification and regression tree, Bayesian modeling averaging

## Abstract

**Background:**

Prostate cancer (PCa) is a common disease in men over 65 years of age, and should be detected early, while reducing unnecessary biopsies. This study aims to construct a classification and regression tree (CART) model (i.e., risk stratification algorithm) using multivariable approach to select Vietnamese men with lower urinary tract symptoms (LUTS) for PCa biopsy.

**Methods:**

We conducted a case-control study on 260 men aged ≥ 50 years who visited MEDIC Medical Center, Vietnam in 2017–2018 with self-reported LUTS. The case group included patients with a positive biopsy and the control group included patients with a negative biopsy diagnosis of PCa. Bayesian Model Averaging (BMA) was used for selecting the most parsimonious prediction model. Then the CART with 5-fold cross-validation was constructed for selecting men who can benefit from PCa biopsy in steps by steps and intuitive way.

**Results:**

*BMA suggested five potential prediction models, in which the most parsimonious model including PSA, I-PSS, and age*. *CART advised the following cut-off points in the marked screening sequence: 18 < PSA < 33.5 ng/mL, I-PSS ≥ 19, and age ≥ 71. Patients with PSA ≥ 33.5 ng/mL have a PCa risk was 91.2%; patients with PSA < 18 ng/mL and I-PSS < 19 have a PCa risk was 7.1%. Patient with 18 ≤ PSA < 33.5ng/mL and I-PSS < 19 have a PCa risk is 70% if age ≥ 71; and is 16% if age < 71. In overall, CART reached high predictive value with AUC = 0.915. Sensitivity, specificity, positive predictive value, negative predictive value, and accuracy of CART at the 20% diagnosis probability threshold were 91.5%, 86.2%, 86.9%, 91.2%, and 88.9% respectively; at 80% diagnosis probability threshold were 79.2%, 92.3%, 91.2%, 81.6%, and 85.8% respectively.*

**Conclusion:**

CART combining PSA, I-PSS, and age has practical use in hospital-based PCa screening in Vietnamese men with lower urinary tract symptoms.

## Background

Prostate cancer (PCa) is common in men, especially in those aged 65 years and older. It has the second-highest incidence/prevalence (i.e., 30.7 per 100 000) and ranks fifth in cancer mortality rate among men (i.e., 7.7 per 100 000) worldwide [1]. In Vietnam, the incidence of PCa is 12.2 per 100 000, and the mortality rate is 5.1 per 100 000 as of 2020 [2]. Approximately 95–98% of PCa cases are adenocarcinomas that develop from adrenal duct cells [3]. PCa treatment depends primarily on the stage of development and the cell and patient characteristics. According to the American Cancer Society, PCa patients diagnosed at the localised or regional stage have a 5-year survival rate of over 90%. However, in the distant stage, the survival rate is only 30% [4]. Therefore, PCa should be detected at an early stage.

Prostate-specific antigen (PSA) is a serine protease in the kallikrein family and considered a tool for the screening and early detection of PCa [5]. It can help detect as early as nine years before having clinical symptoms [6]. There are two types of PCa screening studies using PSA, including population-based and hospital-based (or opportunistic) screenings [7]. The first type of screening deals with testing asymptomatic men with only PSA, and those with elevated PSA are immediately referred to biopsy. However, the latter type of screening involves testing men with some symptoms (e.g., lower urinary tract symptoms) using PSA and other clinical tools. Therefore, all men referred for biopsy in population-based screening are at lower risk of having PCa compared to that of hospital-based screening. PSA only based screening could accounted for 45–70% of the reduction in PCa mortality [8]; it could also induce the unnecessary biopsies [9]. In a 16 year follow-up of the European Randomized Study of Screening for Prostate Cancer (ERSPC), the unnecessary biopsy was 76% (i.e., 76% of elevated PSA cases have a negative biopsy) [10]. In addition, the optimal cut-off value of PSA for confirming PCa remains to be determined [11] [5]. In particular, even at a low level of PSA (that is, lower than 4 ng/mL), the false negative rate of PCa was high at 15%, whereas, at a high level of PSA (that is, higher than 10 ng/mL), the false positive rate was 50% [5].

In Vietnam, population-based PCa screening using PSA was conducted 12 years ago; it indicated a low prevalence of PCa (2.5%), but a high rate of medium grade lesions. The author also implied that the benefit of a mass screening program for PCa was not proven. Instead, a selective PCa screening in the usual care and at the hospital was superior in Vietnam. In hospital-based screening, combining clinical parameters, PSA, age, and other risk factors improved the prediction of prostate cancer [12–14]. International Prostate Symptom Score (I-PSS) is a screening scale for lower urinary tract symptoms and is used to screen non-specific prostate gland abnormalities. For PCa screening. For PCa screening, the I-PSS scale showed reasonable sensitivity (78%), but the specificity was not high (59.4%) [15]. A previous study showed that PSA screening performance varied with different I-PSS values. Therefore, combining PSA and I-PSS could improve the screening benefits [16]. There is, however, a paucity of such practical multivariable algorithm for hospital-based PCa screening in Vietnam.

The approach of PCa screening based on machine learning algorithms has only recently been applied. Algorithms including logistic regression, artificial neural networks, random forests, support vector machines, and extreme and light gradient boosting machines have been demonstrated to enhance PCa screening efficiency [13, 17–20]. However, these models do not help make clinical decisions in a step-to-step and intuitive manner. Classification and regression tree (CART) is an approach that allows physicians to apply results of the screening process directly and intuitively [17, 21].

Our study aimed to investigate the association of PSA, I-PSS, epidemiological and behavioural characteristics with PCa and then used these factors to construct a classification and regression tree (CART) algorithm to select Vietnamese men with lower urinary tract symptoms (LUTS) for PCa biopsy. The algorithm is expected to aid in reducing the probability of a negative prostate biopsy (i.e., unnecessary biopsy) while maintaining the ability to reduce PCa mortality for Vietnamese patients.

## Methods

### Study design and setting

We conducted a case-control study at the MEDIC Medical Center, Ho Chi Minh City, Vietnam. MEDIC is the first and top modernity private medical centre in Vietnam. Every day, more than 4,000 patients visit the centre for examination and treatment. The study was approved by the local institutional ethics committee of the MEDIC Center, and the opinion was signed on 15th July, 2016.

### Participants

Our study participants were men aged ≥ 50 years who visited the MEDIC Centre in 2017–2018 with self-reported lower urinary tract symptoms. The inclusion criteria were abnormal lower urinary tract symptoms or enlarged prostate glands identified through DRE or ultrasound images. The exclusion criterions were acute prostatitis or refusal to participate in the study. All patients who meet the selection criteria were prescribed a biopsy. The case group was defined as having a positive biopsy result for PCa, and the control was defined as having a negative biopsy result. Biopsy based on 12-core Transrectal Ultrasound Guided Biopsy of the Prostate [22]. All patients provided written informed consent before participating in the study.

### Sample size

The minimum sample size estimated for each group of case-control studies was 116 patients to provide 90% power and 5% type I error to detect an odds ratio of 2.5. In Vietnam, PSA ≥ 10 ng/mL is considered as the high-risk group of PCa. Therefore, we chose the proportion of PSA ≥ 10 ng/mL equal to 23% in the control group as the proportion of controls with exposure in the sample size formula [23]. Our studies selected 130 patients for each group to ensure larger than the minimum sample size, hence the total patients was 260.

### Data collection and variables’ definition

We collected epidemiological and behavioural characteristics through interviews using a questionnaire and collected clinical and subclinical information from medical records. Epidemiological characteristics included age, number of children, overweight/obesity (BMI ≥ 23 kg/m^2^ [24]), family history of PCa, existence of urinary tract diseases, history of urinary surgery, benign prostate hyperplasia, and exposure to agrochemicals. Lifestyle behaviours included physical activities (≥ 150 min/week for moderate or vigorous intensity [25]), current tobacco smoking, and heavy drinking (binge drinking (i.e., five drinks or more per occasion) on five or more days in the past month [26]). Food consumption behaviours were determined by the frequency of different food consumption types, including red meat, fruits, vegetables, nuts, vegetable oil, tea, and coffee.

International Prostate Symptom Score (I-PSS) was used to assess seven lower urinary tract symptoms: incomplete emptying, frequency, intermittency, urgency, weak stream, straining, and nocturia. I-PSS Vietnamese version was published by the Vietnamese Ministry of Health and recommended for Benign Prostatic Hyperplasia assessment. Each item of I-PSS was classified on a zero to five scale, reflecting the severity of each symptom [27].

Epidemiological and behavioural characteristics and I-PSS were assessed by only one oncologist for consistency. The oncologist was trained for conducting interviews before joining the study. The questionnaire consisted of ten interviews in a pilot sample for structure and content adaptation.

Serum PSA was quantified by a 2-step immunoassay using light-emitting microparticle technology (CMIA) with Alinity CiCi (Abbott) testing machine system. The testing machine system was calibrated, and quality control was performed at least once every day or when changing the reagent batch [28].

### Statistical analysis

#### Descriptive analysis

Frequency and percentage were used to describe qualitative variables, including overweight/obesity, family history of PCa, existence of urinary tract diseases, history of urinary surgery, benign prostate hyperplasia, and exposure to agrochemicals, lifestyle behaviour, and food consumption behaviour. The median and quartiles were used to describe quantitative variables, including I-PSS, PSA concentration, and age. All descriptive analyses were stratified by the case and control groups.

#### Univariable logistic regression

A univariable logistic regression model was used to screen independent variables that were likely to be associated with PCa. The I-PSS score, PSA concentration, epidemiological characteristics, lifestyle behaviour, and food consumption behaviour were tested for association with PCa.

#### Bayesian model averaging (BMA) for model prediction

A BMA approach was used to search for the most parsimonious model for PCa prediction (i.e., minimum explanatory variables and maximum discrimination power) using PSA, I-PSS, epidemiological and behavioural variables. In summary, if there is *n* variables, there will be 2^*n*^ possible models constructing from *n* variables (not including interactive terms). BMA will construct all possible parsimonious prediction models based on the Bayesian Information Criteria (BIC), and posterior probabilities of these models. The final model with the high practical use in the clinical setting can be chosen based on BMA suggesting and clinical considerations.

#### CART model for PCa screening

CART was performed using the rpart function in the *rpart* package, R language (version 4.0.3). Five-folds cross validation was used to training and testing CART model. All independent variables became CART input in this process. The CART pruning was controlled by the maximum depth of the tree set to 4 to construct a reasonable complexity, the minimum number of observations was allowed to be 10 at each node to ensure sufficient supporting data. Diagnosis values of CART including sensitivity, specificity, positive predictive value, negative predictive value, and accuracy (1 – misclassification error) at the 20%, 50% and 80% probability cut-off were extracted.

## Results

### Association of epidemiological and behavioural characteristics with prostate cancer

There were total 260 patients (130 in case group vs. 130 in control group) included in the study. The median age of cases was significantly higher than the controls (71 vs. 61). Based on univariable logistic regression, the risk factors of PCa included age, exposure to agricultural chemicals. Physical exercise and fruit consumption were noted as protective factors of PCa (Table 1).

**Table 1:** Association of epidemiological and behavioural characteristics with Prostate cancer – univariable logistic regression

	Cases (n = 130) *No. (%)*	Controls (n = 130) *No. (%)*	OR (95% CI)	p-value
**Epidemiological characteristics**				
Age *(median [25–75 percentile])*	71 (64–78)	66 (61–71)	1.07 (1.04–1.11)	< 0.001
Overweight or Obesity *(BMI ≥ 23)*	63 (48.5)	49 (37.7)	1.55 (0.92–2.63)	0.103
Family history of Prostate cancer *(yes)*	2 (1.5)	5 (3.9)	0.39 (0.04–2.45)	0.447
Existing of urinary tract diseases *(yes)*	17 (13.1)	20 (15.4)	0.82 (0.39–1.76)	0.723
History of urinary surgery *(yes)*	10 (7.8)	12 (9.2)	0.83 (0.31–2.18)	0.824
Benign Prostate Hyperplasia *(yes)*	14 (10.8)	24 (18.5)	0.53 (0.24–1.14)	0.113
Exposed to agrochemicals *(yes)*	40 (30.8)	20 (15.4)	2.44 (1.29–4.73)	0.005
**Lifestyle behaviour**				
Physical activity *(yes)*	68 (52.3)	86 (66.2)	0.65 (0.33–0.95)	0.032
Current tobacco smoking *(yes)*	62 (47.7)	48 (36.9)	1.56 (0.92–2.64)	0.103
Heavy drinking *(yes)*	14 (10.8)	12 (9.2)	1.19 (0.53–2.67)	0.680
**Food consumption behaviour**				
Red meat *(≥ 3 times/week)*	107 (82.3)	106 (81.5)	1.05 (0.53–2.08)	1.000
Fruits *(≥ 3 times/week)*	65 (50.0)	83 (63.9)	0.57 (0.33–0.96)	0.033
Vegetables *(≥ 3 times/week)*	111 (85.4)	120 (92.3)	0.49 (0.19–1.16)	0.114
Nuts *(≥ 3 times/week)*	9 (6.9)	9 (6.9)	1.00 (0.34–2.95)	1.000
Vegetable oil *(≥ 3 times/week)*	104 (80.0)	95 (73.1)	1.47 (0.79–2.75)	0.242
Tea *(≥ 3 times/week)*	56 (43.1)	53 (40.8)	1.10 (0.67–1.80)	0.706
Coffee *(≥ 3 times/week)*	82 (63.1)	80 (61.5)	1.07 (0.63–1.82)	0.898

### Association of I-PSS and PSA with prostate cancer

The PCa odds ratio for 1 ng/mL PSA increase was 1.06 (95% CI, 1.05–1.08). The PCa odds ratio for each I-PSS point increase was 1.14 (95% CI, 1.09–1.18). All the items of I-PSS significantly associated with PCa, except for the “Straining” item with lowest OR = 1.16 (95% CI, 0.98–1.38). “Nocturia” item had highest OR with 2.22 (95% CI, 1.79–2.74), and “Urgency” item came to the second with OR = 1.41 (95% CI, 1.23–1.62) (Table 2).

**Table 2:** Association of I-PSS and PSA with prostate cancer - univariable logistic regression

	Cases (n = 130) *Median* *(25–75 percentile)*	Controls (n = 130) *Median* *(25–75 percentile)*	OR (95% CI)	p
**PSA concentration (ng/mL)**	89.5 (39.1–100)	12 (8–19)	1.06 (1.05–1.08)	< 0.001
**I-PSS (total score)**	***14 (8–19)***	***6.5 (3–12)***	***1.14 (1.09–1.18)***	***< 0.001***
Incomplete Emptying	4 (1–5)	1 (1–5)	1.27 (1.12–1.44)	
Frequency (every 2 h)	2 (0–2)	0 (0–3)	1.22 (1.08–1.37)	
Intermittency	0 (0–1)	0 (0–1)	1.29 (1.02–1.64)	
Urgency	2 (0–5)	0 (0–1)	1.41 (1.23–1.62)	
Weak Stream	0 (0–2)	0 (0–0)	1.32 (1.12–1.55)	
Straining	0 (0–1)	0 (0–1)	1.16 (0.98–1.38)	
Nocturia	4 (3–5)	2 (1–3)	2.22 (1.79–2.74)	

### BMA for PCa prediction

To determine whether PCa could be predicted by PSA, I-PSS, epidemiological and behavioural variables. There were 27 models suggested from BMA process, among them the best 5 models are shown in Table 3. The most parsimonious model (i.e., minimum explanatory variables and maximum discrimination power) included two variables: I-PSS, and PSA concentration. The second parsimonious model contained I-PSS, PSA, and age. The Area Under the ROC Curve (AUC) of the most parsimonious model was not much different compared to the second parsimonious model (0.931 vs. 0.929). Because age is an important factor for PCa screening and diagnosis in many previous studies [29–31], it is also a critical factor for disease mechanisms from a clinical standpoint. Therefore, we chose the second model with three variables as the best model to use in the clinical setting (Table 3). This final model is also in light with the final model suggested by CART algorithm (details shown below).

**Table 3:** BMA prediction models using I-PSS, PSA, epidemiological, and behavioural characteristics for PCa

Model	Variable	OR (95% CI)	p	R^2^ (%)	AUC	BIC	Posterior probability
1	IPSS	1.12 (1.06–1.18)	< 0.001	49.2	0.931	199.9	0.364
	PSA	1.06 (1.04–1.08)	< 0.001				
	Intercept	0.03 (0.01–0.08)	< 0.001				
2	IPSS	1.11 (1.05–1.17)	< 0.001	50.3	0.929	201.4	0.174
	PSA	1.06 (1.04–1.07)	< 0.001				
	Age	1.06 (1.01–1.10)	0.047				
	Intercept	0.01 (0.00–0.04)	< 0.001				
3	IPSS	1.13 (1.07–1.19)	< 0.001	50.1	0.929	205.5	0.120
	PSA	1.06 (1.04–1.08)	< 0.001				
	Fruits *(≥ 3 times/week)*	0.50 (0.23–1.06)	0.069				
	Intercept	0.05 (0.02–0.12)	< 0.001				
4	IPSS	1.12 (1.06–1.19)	< 0.001	49.7	0.931	203.6	0.057
	PSA	1.06 (1.04–1.08)	< 0.001				
	Overweight or Obesity	1.68 (0.80–3.53)	0.174				
	Intercept	0.03 (0.01–0.07)	< 0.001				
5	IPSS	1.12 (1.06–1.18)	< 0.001	49.7	0.931	203.7	0.056
	PSA	1.06 (1.04–1.08)	< 0.001				
	Vegetable oil *(≥ 3 times/week)*	1.84 (0.75–4.52)	0.186				
	Intercept	0.02 (0.01–0.07)	< 0.001				

### CART for PCa screening

**Fig. 1:**
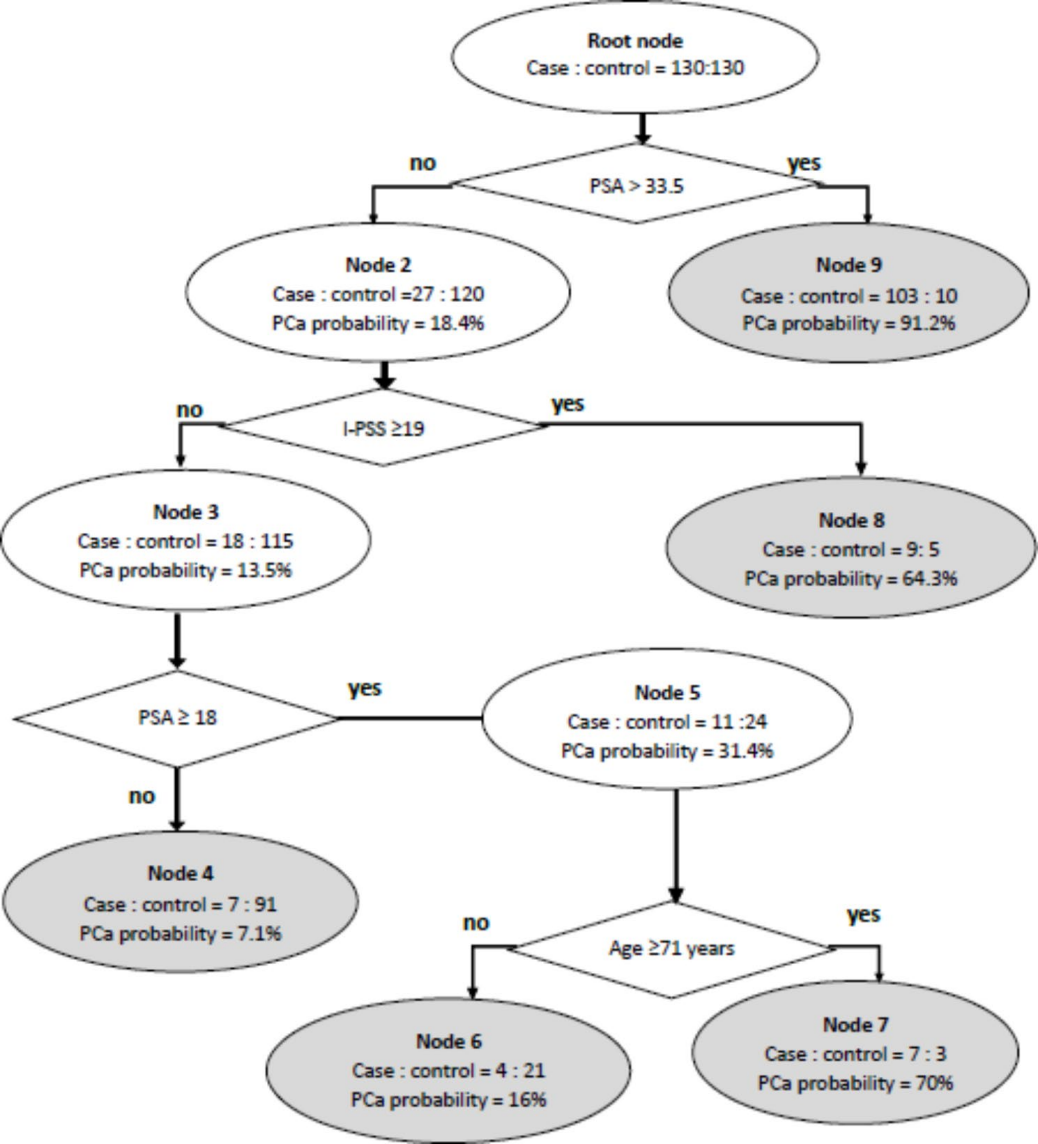
Trained CART in prostate cancer screening

CART was deployed with all independent variables input for PCa screening, and the final model is shown in (Fig. 1).

The results indicated that PSA, I-PSS, and age played important roles in PCa screening. CART advised the following cut-off points in the marked screening sequence: 18 < PSA < 33.5 ng/mL, I-PSS ≥ 19, and age ≥ 71. Patients with PSA ≥ 33.5 ng/mL have a PCa risk was 91.2%; patients with PSA < 18 ng/mL and I-PSS < 19 have a PCa risk was 7.1%. Patient with 18 ≤ PSA < 33.5ng/mL and I-PSS < 19 have a PCa risk is 70% if age ≥ 71; and is 16% if age < 71.

In overall, CART reached high predictive value with AUC = 0.915. Sensitivity, specificity, positive predictive value, negative predictive value, and accuracy of CART at the 20% diagnosis probability threshold were 91.5%, 86.2%, 86.9%, 91.2% and 88.9% respectively; at 80% diagnosis probability threshold were 79.2%, 92.3%, 91.2%, 81.6%, and 85.8% respectively (Table 4).

**Table 4:** Diagnosis values of CART in PCa screening

	Probability = 20%	Probability = 50%	Probability = 80%
Sensitivity	0.915	0.915	0.792
Specificity	0.862	0.862	0.923
Positive predictive value	0.869	0.869	0.912
Negative predictive value	0.912	0.912	0.816
Accuracy	0.889	0.889	0.858

## Discussion

### Epidemiological and behavioral characteristics

The study included 260 observations at the Medic Center HCMC with 130 in the case group and 130 in control group. Based on univariable logistic regression, the risk factors of PCa included age, exposure to agricultural chemicals and protective factor included physical exercise and fruit consumption.

Previous studies found that farming and exposure to agricultural chemicals are risk factors for PCa but not for all agricultural chemicals [29, 30]. Exposure to a few specific pesticides including fonofos, malathion, terbufos, and azinphos-methyl, dimethoate associated with PCa [31–33]. Genomic analysis showed pesticides might interact with genetic variants in pathways related to neurotransmission release in PCa patients [34, 35]. Therefore, the relationship between exposure to agricultural chemicals and PCa is plausible. Further epidemiological and mechanism studies are needed to identify the relationships of PCa with specific agricultural chemicals, in particular in the Vietnamese context.

Although our study initially found a relationship between physical exercise and PCa, there is a lack of evidence in the literature. Recent review and meta-analyses reveal that the association between regular physical activity and a low risk of prostate cancer remains elusive [36, 37]. Given also many general health benefits of physical activity, there is the need to clarify the role of physical activity in association with PCa in further studies.

The association of fruit consumption with PCa was shown in our study and recent studies [38, 39]. Total fruit intake significantly reduced PCa risk. However, our study did not analyze fruit subtypes. A previous study found that citrus fruit consumption is associated with PCa, but other fruit subtypes are not associated [38]. This relationship might be due to the anti-carcinogenic properties of vitamins and phytochemicals in citrus fruits [40, 41]. However, the causal relationship remains unclear because most findings are based on a cross-sectional study.

CART model suggested that age is most important in epidemiological and behavioral characteristics. Therefore, age, PSA, and IPSS were combined in PCa screening CART model. Our study showed that 50% of patients were older than 71 years in cases and older than 66 in controls. PCa is a disease that commonly occurs in the elderly men. Previous studies found that 75–80% of new cases occur in men aged over 65 years [42, 43]. Another study in the United States had an average participant’s age was 66 years [44]. A study by European Association of Urology showed that PCa rarely occurred in men younger than 50 years; it also indicated that the median age of PCa patient was 70 years [45]. Giwercman et al. showed that age was the closest risk factor of PCa [46]. Similarly, our study detected age as an independent risk factor of PCa. According to the Bayesian Model Averaging process, the PCa risk increased by 6% each year of age increased.

***The role of I-PSS in PCa screening***.

The Prostate Symptom Score (I-PSS) with seven recommended questions became an international standard to assess the symptoms of urination dysfunction in patients during the previous month. This scale is able to monitor changes in symptoms over time or after an intervention. A symptom severity assessment with an I-PSS scale is an important part of the initial evaluation, diagnosis, prediction, and monitoring of response to treatment [47, 48]. Our study noted that I-PSS was used to detect the symptoms of PCa (p < 0.001) in both univariable and multivariable analyses. A cohort study by Martin et al. [49] detected an association between I-PSS and PCa. For overall PCa, men with I-PSS ≥ 20 had a 2.26-fold increased risk of PCa compared to those with no symptoms. For localised PCa, men with I-PSS ≥ 20 had a 4.6-fold increased risk of PCa compared to those with no symptoms [49]. A study by Hosseini et al. [15] showed an association between I-PSS and PCa. The mean I-PSS score of the PCa group was 16.05 and higher than that of the non-PCa group, with a mean I-PSS score of 6.84. The prevalence of patients with I-PSS ≥ 20 in the PCa group was 30.3%, which was higher than that in the non-PCa group. The sensitivity and specificity of I-PSS at cut-off I-PSS ≥ 20 were 78% and 59%, respectively [15]. Our study and data have provided evidence about the relationship of PCa screening value with I-PSS. In Vietnam, according to the Ministry of Health, I-PSS has not yet been recommended for PCa initial screening; however, I-PSS has been recommended for benign hypertrophy of prostate – a disease that has many symptoms similar to early-stage PCa symptoms. Our study recommended using I-PSS for initial screening for any patient who has self-reported lower urinary tract symptoms.

#### The role of PSA in PCa screening

Prostatic specific antigen (PSA) is an antigens-proteolytic protein that is secreted by prostate cells and excreted into the glandular microducts, which are largely poured into the sperm through the crystalline ducts, and smaller portions are poured into the serum and lymphatic secretions. PSA increases in PCa, prostate benign proliferation, and prostate inflammation after the procedure (cystoscopy, catheterisation of urethral, prostate massage, after a prostate biopsy within 4 weeks, after ejaculation within 48 h). PSA decreases by 50% when taking 5 alpha-reductase inhibitors with a continuous period of over 6 months [50].

In our study, PSA shown a significant associated with PCa and is an important predictor for PCa in both BMA and CART algorithm. Currently, all guidelines of the American and European Urogenital Societies use PSA cut-off levels ranging from 2 ng/mL to 4 ng/mL in order to make prostate biopsy decision [51]. Meanwhile, researchers chose PSA > 4 ng/mL as the cut-off level to ensure high sensitivity in screening [52–54]. According to Vietnam Ministry of Health guideline, PSA > 4 ng/mL has been recommended for selecting patients with lower urinary tract symptoms for a further clinical assessment for PCa diagnosis. The cut-off value of PSA for referring biopsy, however, is not determined. Previous studies showed that only using PSA for PCa screening before biopsy could tend to the high probability of a negative biopsy out of elevated PSA cases (high proportion of unnecessary biopsy). In PCa patients, only 65–75% of cases have PSA > 4 ng/mL; 35% of the remaining PSA cases remain at a normal level [55]. The study by Thompson et al. [56] in U.S. on cancer screening with 2950 men over 50 years old showed that 15.2% of patients had PSA < 4 ng/mL got prostate cancer, as well as 14.9% of the negative prediction group with a Gleason score of $\ge$7 [56]. Wright et al. [57] found that a PSA threshold of > 4 ng/mL detected more cancer cases but increased unnecessary biopsy cases [57]. Morgan et al. [28] noted that the sensitivity of reached 98.2% at PSA cut-off level of > 4 ng/mL, and the sensitivity at PSA > 10 ng/mL was 91% with a specificity of 54% [28]. In some cases, the serum PSA values in the PCa and non-PCa groups overlapped, especially when PSA levels were 4–10 ng/mL. The PSA value in this range was called “diagnostic gray zone,“ according to Shariat and Karakiewicz [58].

In hospital-based PCa screening, combining PSA, clinical parameters, age, and other risk factors could reduce the rate of unnecessary biopsy while maintaining the ability to reduce PCa mortality [12–14].

### CART value for PCa screening

To remedy the inherent limitations of PSA in PCa screening, we used a combination of PSA with I-PSS and the main risk factors of PCa to build the CART model. Based on CART, patients with a PSA cut-off level > 33.5 ng/mL have a PCa risk of up to 91.2%. Patients with I-PSS < 19, and PSA < 18 ng/mL were at 7.1% risk. CART overcomes the limitations of using only I-PSS or PSA for screening. Other machine learning algorithms have been used in PCa screening in previous studies and have reached higher values than PSA only. In a study by Babaian et al. [59], a neural network algorithm for PCa screening showed an improved value compared to using only PSA, the specificity of the neural network was not good (lower than 65%) [59]. A study by Satoshi et al. [17] showed that artificial neural network, random forest, and support vector machine improved overall value when compared to only PSA; however, sensitivity and specificity were usually lower than 80% [17]. Our CART algorithm with three variables, PSA, I-PSS, and age, showed a relatively high predictive power (AUC = 0.915). In addition, CART algorithm could also support physicians to make clinical decisions in a step-to-step and intuitive manner; hence it has practical use in a daily clinical setting. At 20% diagnosis probability threshold, CART showed a high negative predictive value (91.2%), and at 80% diagnosis probability threshold, CART also had a high positive predictive value (91.2%). Therefore, we recommended 20% diagnosis probability threshold for negative prediction and 80% diagnosis probability threshold for referring prostate biopsy. Any other patients with a probability of PCa range from > 20% to < 80%, further tests including the digital rectal examination (DRE), PSA re-test after a month, and transrectal ultrasonography (TRUS) should be considered to reduce unnecessary biopsy while keeping the ability to diagnose PCa early.

The study has some limitations. First, we lack other tests such as DRE, TRUS, biomarkers that can contribute to making biopsy decisions [5, 7]. Second, the CART model has not yet been tested in different populations for the validity and reliability of the algorithm. Finally, we could not estimate the overdiagnosis rate of PCa in the study. It warrants further study in the near future to overcome these limitations.

## Conclusion

CART advised the following cut-off points in the marked screening sequence: 18 < PSA < 33.5 ng/mL, I-PSS ≥ 19, and age ≥ 71. Patients with PSA ≥ 33.5 ng/mL have a PCa risk was 91.2%; patients with PSA < 18 ng/mL and I-PSS < 19 have a PCa risk was 7.1%. Patient with 18 ≤ PSA < 33.5ng/mL and I-PSS < 19 have a PCa risk is 70% if age ≥ 71; and is 16% if age < 71. In overall, CART reached high predictive value with AUC = 0.915. Sensitivity, specificity, positive predictive value, negative predictive value, and accuracy of CART at the 20% diagnosis probability threshold were 91.1%, 86.9%, 86.2%, 91.5% and 88.9%; at 80% diagnosis probability threshold were 81.6%, 91.2%, 92.3%, 79.2%, and 85.8%. I-PSS, PSA, and age had importance role in PCa screening. CART combining PSA, I-PSS, and age has practical use in hospital-based PCa screening in Vietnamese patients.

## Data Availability

The datasets used and/or analysed during the current study are available from the corresponding author on reasonable request.
